# Poly (γ-Glutamic Acid) Promotes Enhanced Dechlorination of *p*-Chlorophenol by Fe-Pd Nanoparticles

**DOI:** 10.1186/s11671-018-2634-y

**Published:** 2018-07-24

**Authors:** Shiyu Zhang, Chao Zhang, Mingyue Liu, Renliang Huang, Rongxin Su, Wei Qi, Zhimin He

**Affiliations:** 1 Tianjin Key Laboratory of Indoor Air Environmental Quality Control, School of Environmental Science and Engineering, Tianjin University, Tianjin, 300072 People’s Republic of China; 2 State Key Laboratory of Chemical Engineering, School of Chemical Engineering and Technology, Tianjin University, Tianjin, 300072 People’s Republic of China; 3 Collaborative Innovation Center of Chemical Science and Engineering (Tianjin), Tianjin, 300072 People’s Republic of China; 4 Tianjin Key Laboratory of Membrane Science and Desalination Technology, Tianjin University, Tianjin, 300072 People’s Republic of China

**Keywords:** Poly (γ-glutamic acid), Fe-Pd nanoparticles, Zero-valent iron, *p*-Chlorophenol, Environmental remediation

## Abstract

Nanoscale zero-valent iron (nZVI) has shown considerable promise in the treatment of chlorinated organic compounds, but rapid aggregation and inactivation hinder its application. In this study, palladium-doped zero-valent iron nanoparticles involving poly (γ-glutamic acid) (Fe-Pd@PGA NPs) were synthesized. The nanoparticles were small (~100 nm), uniformly distributed, and highly stable. The dechlorination performance of Fe-Pd@PGA NPs was evaluated using *p*-CP as a model. The results demonstrated that Fe-Pd@PGA NPs show high activity even in weakly alkaline conditions. The maximum rate constant reached 0.331 min^− 1^ at pH 9.0 with a Fe to *p*-CP ratio of 100. Additionally, the dechlorination activity of Fe-Pd@PGA NPs is more than ten times higher than that of the bare Fe-Pd NPs, demonstrating the crucial role of PGA in this system. Furthermore, we investigated the dechlorination performance in the presence of different anions. The results indicated that Fe-Pd@PGA NPs can maintain high activity in the presence of Cl^−^, H_2_PO_4_^−^, and humic acid, while HPO_4_^2−^and HCO_3_^−^ ions slightly reduce the dechlorination activity. We believed that PGA is a promising stabilizer and promoter for Fe-Pd NPs and the Fe-Pd@PGA NPs have the potential for practical applications.

**Electronic supplementary material:**

The online version of this article (10.1186/s11671-018-2634-y) contains supplementary material, which is available to authorized users.

## Background

Water pollution has raised global concerns on environmental sustainability and many high-performance materials had been developed for the effective pollution treatments [1–5]. Nanoscale zero-valent iron (nZVI) has been intensively studied and is regarded as one of the most promising nanomaterials for environmental remediation of diversified contaminants, such as chlorinated organic compounds, heavy metal ions, pesticides, and organic dyes [1, 6, 7]. It has the extraordinary ability to transform toxic contaminants into non-toxic forms through chemical reduction processes, but nZVI nanoparticles easily react with water and lose electrons that contributing to the degrading reaction [8]. Therefore, another metal was always used as a dopant to produce a bimetallic nanoparticle, such as Fe-Pd or Fe-Ni, to increase the degradation rates [9, 10]. However, there are van der Waals and attractive magnetic forces between nZVI particles, which lead to significant aggregation, hindering their transport in contaminated aquifers and impairing the reactivity [11, 12]. The intrinsic shortcomings of nZVI, even bimetallic systems, could weaken the effect of removal and limit the feasibility of utilization.

To inhibit aggregation, extensive efforts had been made to apply polymer surface stabilizers (e.g., polysaccharides, polyelectrolytes, and surfactants). These stabilizers could provide both electrostatic and steric repulsion to prevent particle aggregation [13]. For example, He et al. [14] synthesized nZVI stabilized by carboxymethyl cellulose (CMC), and the derived nanoparticles were successfully used for in situ destruction of chlorinated ethenes, such as trichloroethylene (TCE) and polychlorinated biphenyls (PCBs). Petersen et al. [15] produced nZVI in the presence of poly (acrylic acid) (PAA), achieving a high degradation rate for chlorinated contaminants. Liu et al. [16] synthesized CMC-stabilized and anionic polyacrylamide (APAM)-stabilized nZVI and observed that APAM modification led to the aggregation of nZVI in suspension, while CMC modification made nZVI disperse well. This study provided evidence that molecular structure played an important role in the modification effect of nZVI. Many other different types of macromolecular modifiers have been used to coat nZVI particles. [17] In addition to the aggregation problem, another issue is the surface passivation due to the formation of oxide layers, such as goethite (α-FeOOH), maghemite (γ-Fe_2_O_3_), and hematite (α-Fe_2_O_3_). Previous studies demonstrated that acidic conditions favor avoiding the surface passivation of nZVI and keeping its surface activation [18–20]. Therefore, pH value is a crucial parameter that affects oxide layer formation. There have been many reports studying the reaction under acidic conditions to improve dechlorination efficiency [21, 22]. Additionally, rapid inactivation caused by common anions and humic acid (HA, organic matter commonly found in water [23]) also limited the field application of nZVI [24, 25]. Therefore, nZVI-based materials adapted to alkaline conditions with different anions are expected.

Poly (γ-glutamic acid) (PGA), a natural anionic polypeptide produced by Bacillus subtilis, is nontoxic, highly water-soluble, and can be chemically, physically, and enzymatically degraded in nature. It is made of units of l- and d-glutamic acids connected with amides between α-amino and γ-carboxylic acid [26]. It has broad applications in the food, chemical, biological, and medical industries [27–29]. In addition, PGA is a potential biosorbent for the removal and recovery of heavy metals from wastewaters due to its ability to bind metal ions. For example, Jamiu et al. [30] synthesized a PGA-based resin as a sorbent for sequestering Co^2+^ ions from aqueous solution. Hu et al. [31] developed a biocomposite sorbent incorporating PGA for adsorption and recovery of Cu^2+^ ions. Bodnar et al. [32] described the preparation of biodegradable nanoparticles based on the complexation of PGA with Pb^2+^ ions, demonstrating that PGA was a promising sorbent for the removal of heavy metals in polluted water. Taking into consideration that anionic PGA has abundant carboxyl groups, which can provide electrostatic and steric repulsion to prevent particle aggregation, as well as high binding ability toward the metal ions via chelation and electrostatic binding [33, 34], we attempted to use it as a stabilizer and coordination agent to synthesize Fe-Pd NPs and further chelate Fe^2+^/Fe^3+^ ions generated from the corrosion of Fe-Pd NPs. We speculate that the PGA can improve the performance of Fe-Pd NPs under alkaline conditions with different anions.

Herein, we synthesized the PGA-modified Fe-Pd nanoparticles (denoted as Fe-Pd@PGA NPs). The nanoparticles were characterized by scanning electron microscopy (SEM), transmission electron microscopy (TEM), X-ray diffraction (XRD), and Fourier transform infrared spectroscopy (FTIR). The colloidal stability of Fe-Pd@PGA NPs was also assessed. Furthermore, *p*-Chlorophenol (*p*-CP) (one of the most toxic phenolic derivatives [35–37]) was selected as a model pollutant to evaluate the dechlorination activity of Fe-Pd@PGA NPs. The dechlorination of *p*-CP over the NPs with different dosages of PGA and Pd loading was investigated. It is worth mentioning that all the experiments were conducted in weakly alkaline conditions without pH control (the pH value was approximately 9). Additionally, we also investigated the dechlorination of *p*-CP in the presence of different anions and HA to evaluate their potential use in real wastewater.

## Methods

### Materials

PGA (M_W_ 100,000–50,000 kDa) was purchased from Yuanye Bio-Technology (Shanghai, China). Ferrous sulfate heptahydrate (FeSO_4_·7H_2_O, > 99.0%), potassium borohydride (KBH_4_, 97%), potassium chloropalladite (K_2_PdCl_4_, 98%), and *p*-chlorophenol (*p*-CP, 99%) were supplied by Aladdin Reagent Company (Shanghai, China). Methanol (CH_3_OH, HPLC grade, 99.9%) was purchased from Concord Technology (Tianjin, China). Sodium chloride (NaCl, 99%), sodium bicarbonate (NaHCO_3_, 99.7%), disodium hydrogen phosphate (Na_2_HPO_4_, 99%), and sodium dihydrogen phosphate (NaH_2_PO_4_, 99%) were purchased from Chemregeant (Tianjin, China). Humic acid (HA, technical) was purchased from Heowins (Tianjin, China). All chemicals were used without further purification. All water was purified by a Sartorius arium pro VF water purification system (18.2 MΩ resistivity).

### Preparation of Fe-Pd@PGA NPs

The Fe-Pd@PGA NPs were synthesized using a chemical reduction method in which potassium borohydride served as a reduction reagent. Then, 250 mg of FeSO_4_·7H_2_O was added into 50 mL of PGA solution containing different mass of PGA (10, 25, 50, and 70 mg), resulting in the desired concentrations of Fe^2+^ and PGA (wt.%). The mixture was then purged with argon for approximately 20 min to assure the formation of Fe^2+^-PGA complex and to remove dissolved oxygen (DO). Then, 3 mL of KBH_4_ (50 mg mL^− 1^) was injected into the mixture at a BH_4_-to-Fe^2+^ molar ratio of 3.0 to form the Fe NPs. To ensure the efficient use of the reductant, the suspension was purged with Argon for 15 min until gas (hydrogen) evolution ceased. Then, Fe nanoparticles were either tested as monometallic particles or loaded with trace amounts (0.1, 0.2, 0.4, and 0.8 wt.% Pd/Fe) of Pd to yield the PGA-stabilized bimetallic nanoparticles by adding known quantities of potassium chloropalladite aqueous solution into the nanoparticle suspension. In addition, bare Fe-Pd NPs with different degrees of Pd loading were also prepared for comparison. The system was constantly purged with argon during the synthesis and dechlorination reaction. The temperature of the solution was kept at 25 °C by a thermostatic water bath.

### Stability Test

For the sedimentation experiments, Fe-Pd@PGA NPs suspensions prepared in the last step were transferred from the reactor into a 50 mL glass tube. The suspension was shaken without sonication before the experiment. Then, the 50 mL glass tube was allowed to settle, and the sedimentation extent was recorded by taking photos of the glass tubes at periodic intervals.

### Batch Dechlorination Experiments

The 550 mg L^− 1^ stock solution of *p*-CP was prepared by dissolving *p*-CP in water at 25 °C. Further, 2 mL of *p*-CP stock solution was added to the prepared Fe-Pd suspension to result in the desired initial molar ratio of Fe to *p*-CP (105:1) and initial *p*-CP concentration of 20 mg L^− 1^. Then, the degradation of *p*-CP was initiated and the reaction was carried out with the argon purge. For the measurement of the concentration of *p*-CP, 0.8 mL of liquid sample was taken off from the solution at desired selected times, then a magnet was used to speed up the sedimentation. The liquid supernatant was then filtered through a syringe membrane filter (0.22 μm) and further tested with high-performance liquid chromatography (HPLC). All the experimental points were duplicated in two consecutive experiments, and the standard deviations were calculated.

### *p*-CP Removal Tests in Simulated Groundwater

In these tests, the initial concentration of *p*-CP was set to 20 mg L^− 1^, the same as the previous concentration of batch dechlorination experiments, anion concentrations were set to 1 mM, and 5 mg/L humic acid was added to the suspension to simulate a naturally occurring groundwater situation. The simulated groundwater recipe is composed of different kinds of anions after adding different kinds of inorganic salts, including NaCl (0.1 mM), Na_2_HPO_4_·12H_2_O (0.1 mM), NaH_2_PO_4_ (0.1 mM), and NaHCO_3_ (1 mM). The pH of reaction suspension was also recorded during the reaction.

### Characterization

Scanning electron microscopy (SEM, Hitachi S-4800) and high-resolution transmission electron microscopy (HRTEM, JEM-2100F) were used to investigate the morphology and size of the bare and PGA modified Fe-Pd NPs. The HRTEM samples were diluted and ultrasonicated in ethanol, followed by dropping the resulting suspension on the carbon-coated copper grids and drying at room temperature. X-ray diffraction (XRD) measurement was carried out using a Bruker D8-Focus measuring instrument with a Cu Kα X-ray source. Fourier transform infrared spectroscopy (FTIR, Nicolet Nexus 670) analyses were conducted to elucidate the interaction between the Fe-Pd NPs and PGA. The samples mentioned before for the “Characterization” section were washed with water and alcohol three times, then dried in a vacuum oven at room temperature and stored. The *p*-CP concentration in solution was measured by HPLC.

## Results and Discussion

### Synthesis and Characterization of Fe-Pd@PGA NPs

**Scheme 1:**
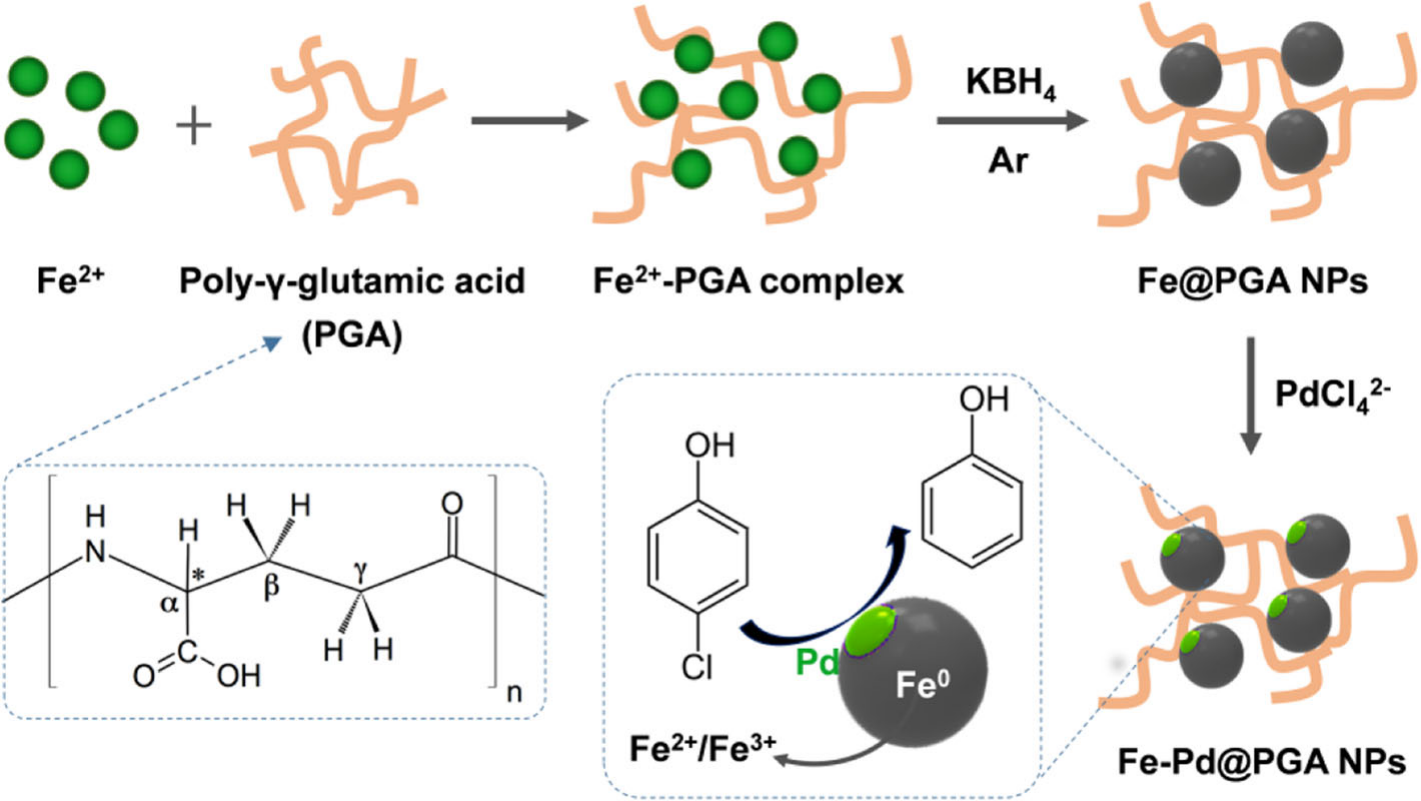
Schematic illustration for the synthesis of Fe-Pd@PGA NPs and the dechlorination of *p*-CP

As shown in Scheme 1, zero-valent Fe NPs (Fe@PGA NPs) were synthesized by reducing Fe^2+^ with KBH_4_ in the presence of PGA. After that, Fe-Pd@PGA NPs were obtained via the replacement reaction between Pd^2+^ (K_2_PdCl_4_) and Fe^0^. To evaluate the dechlorination activity, *p*-CP was selected as a model pollutant, and the dechlorination process is also shown in Scheme 1.

**Fig. 1:**
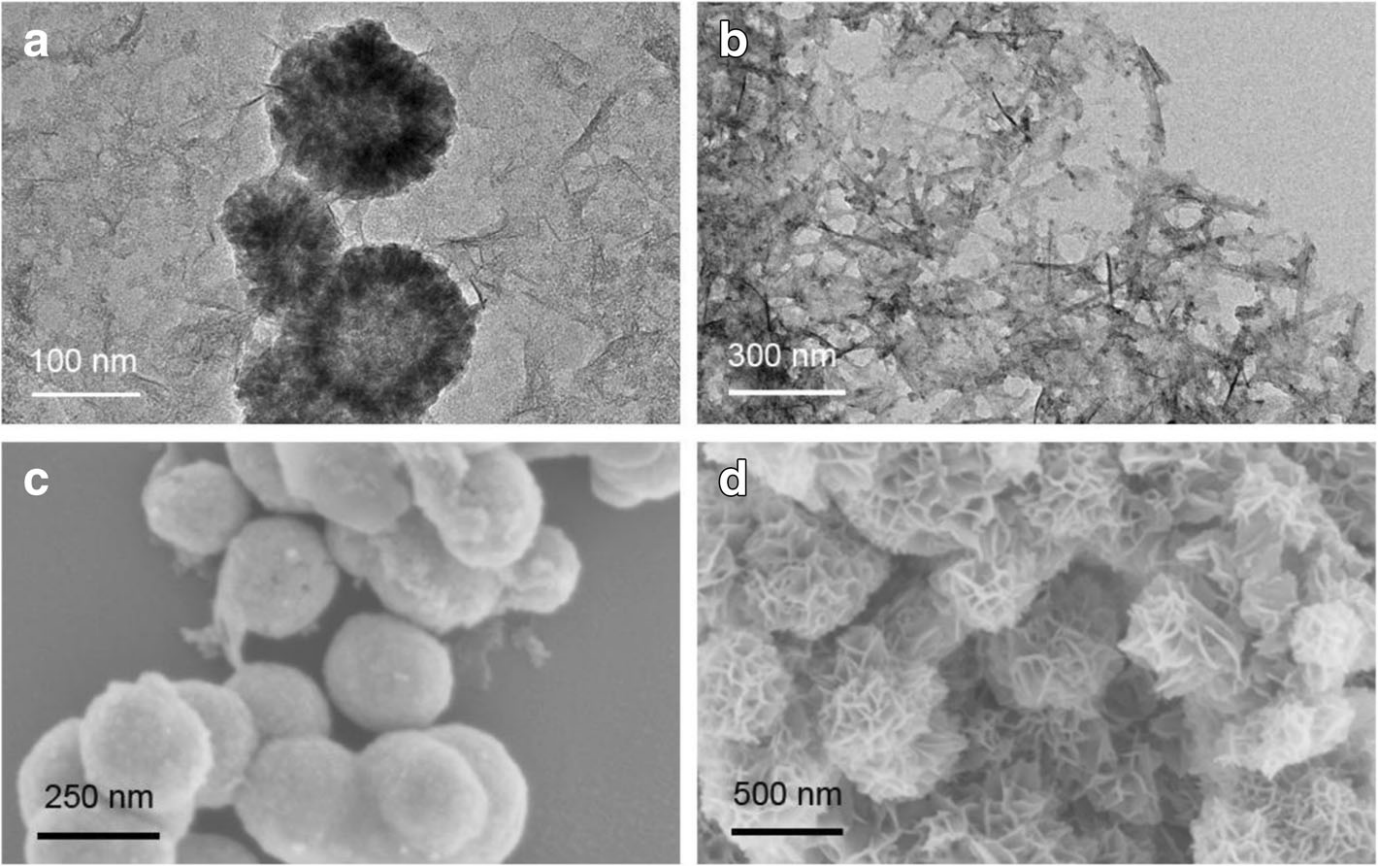
**a**, **b** TEM and **c** SEM micrographs of the Fe-Pd@PGA NPs. **d** SEM micrograph of bare Fe-Pd NPs

The morphologies and size of bare Fe-Pd and Fe-Pd@PGA NPs were characterized by SEM and TEM. As shown in Fig. 1a, b, the majority of Fe-Pd@PGA NPs had relatively spherical shapes in the size range of 50–200 nm and were connected to some chip-like structures, which was ascribed to PGA modification. The structure is similar to that previously reported using the chemical reduction method [38]. The elemental distribution and composition was shown in Additional file 1: Figure S1 and Additional file 1: Table S1. The SEM image shows that the Fe-Pd@PGA NPs had smooth spherical surfaces, while the bare Fe-Pd NPs had already been oxidized, which proved that PGA could inhibit the aggregation and restrain oxidation (Fig. 1c, d). In general, compared with nZVI modified by other stabilizers in a previous work [16]. Fe-Pd@PGA NPs had a similar shape and size.

**Fig. 2:**
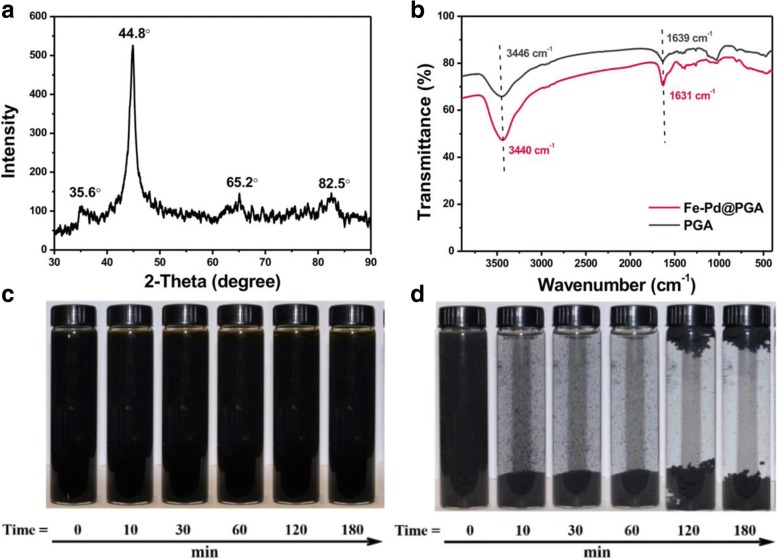
**a** XRD pattern of Fe-Pd@PGA NPs. **b** FTIR spectra of PGA and Fe-Pd@PGA NPs. **c**, **d** Photos of Fe-Pd@PGA NPs (**c**) and bare Fe-Pd NPs (**d**) suspension at different times

Figure 2a shows the XRD pattern of Fe-Pd@PGA NPs that had settled in air for 1 day. The peak at 2θ of 44.8^°^ and a weak peak at 82.5^°^ indicated the presence of α-Fe^0^ with a poorly ordered and amorphous nature in the NPs [16, 39]. Another two weak characteristic peaks appeared at 2θ of 35.6^°^ and 65.2^°^, which matched the corrosion products of Fe^0^, magnetite (Fe_3_O_4_), and/or magnetite (γ-Fe_2_O_3_) [40, 41], suggesting that only a small portion of Fe^0^ was oxidized. The XRD pattern verified that nZVI had been created successfully.

Figure 2b is the FTIR spectra of PGA and Fe-Pd@PGA NPs in the range of 4000–400 cm^− 1^. The absorption peak at 3440 cm^− 1^ is a characteristic of the O − H stretching vibration of the hydroxyls in the carboxyl groups. The absorption peak in the vicinity of 1631 cm^− 1^ is a characteristic of the C=O stretching vibration of carboxyl groups. The absorption peak between 900 and 650 cm^− 1^ comes from the amido groups of PGA [42, 43]. The slight shift of the absorption peak of the Fe-Pd@PGA NPs indicated the interaction of the Fe-Pd NPs and PGA.

The colloidal stability of Fe-Pd@PGA NPs and bare Fe-Pd NPs was investigated by sedimentation experiments. The bare Fe-Pd NPs aggregated quickly within 10 min, and the visible black sediment can be observed in the bottom of the tube (Fig. 2d). After 120 min, the flotage and generated flocculation formed, which might be attributed to the generation of hydrogen. As expected, the Fe-Pd@PGA NPs were dispersed well and hardly settled down to the bottom of glass tube for more than 180 min as shown in Fig. 2c. Additionally, we found that the aggregation of Fe-Pd@PGA NPs occurred after standing for 1 day (Additional file 1: Figure S2). The colloidal stability of Fe-Pd@PGA NPs is comparative and even better than the classical CMC-modified Fe-Pd NPs (e.g., for 5 g L^− 1^ of CMC-nZVI, the settlement time is 30 min [44]).

### Dechlorination of p-CP by Fe-Pd@PGA NPs

**Fig. 3:**
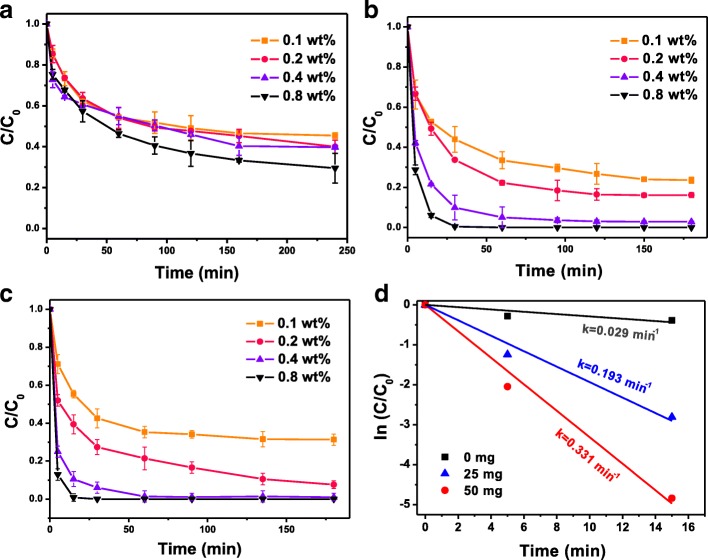
**a**–**c** Time courses of dechlorination using bare Fe-Pd NPs (**a**) and Fe-Pd@PGA NPs (**b**, **c**) with different PGA loadings (**b** 25 mg PGA; **c** 50 mg PGA) and different Pt loadings ranged from 0.1 to 0.8 wt.%. **d** The rate constant (*k*) for the dechlorination of *p*-CP with different PGA loadings at pH 9.0 (Pd loading = 0.8 wt.%)

The dechlorination activity of the Fe-Pd@PGA NPs was investigated through batch *p*-CP dechlorination tests at weakly alkaline conditions (pH = 9). Figure 3a–c shows the removal of *p*-CP using bare Fe-Pd and Fe-Pd@PGA NPs with different Pd loadings. The results indicated that the bare Fe-Pd NPs without PGA modification could only remove approximately 50% *p*-CP in 4 h, while Fe-Pd@PGA NPs have a largely enhanced degradation efficiency. Specifically, Fe-Pd@PGA NPs with 25 mg PGA and 0.8% Pd loading could remove over 90% *p*-CP within 30 min. A further increase in PGA loading to 50 mg could achieve 100% *p*-CP removal within 30 min. These results demonstrated that PGA promotes the enhanced dechlorination activity of Fe-Pd NPs. From the perspective of a surface-mediated reaction, the reactivity was proportional to the available surface area [39]. PGA modification could prevent the nanoparticles from agglomerating, then Fe-Pd NPs were smaller and consequently had larger total surface area per unit mass. As expected, Fe-Pd@PGA NPs were more effective than the bare Fe-Pd NPs for removing *p*-CP.

During the dechlorination of *p*-CP by Fe-Pd@PGA NPs, the Pd loading is also an important factor for the dechlorination activity. As shown in Fig. 3b, c, the Fe-Pd@PGA NPs with higher Pd content displayed higher dechlorination activity. This phenomenon is consistent with that reported in many previous studies. An explanation is that Fe and Pd can form galvanic cells, and Pd could accept electrons from Fe, which played catalytic roles not only accelerating Fe corrosion but also improving dechlorination activity [45]. It is worth noting that, without PGA modification, it is difficult to achieve high dechlorination efficiency even with high Pd loading. Therefore, the modification of PGA is crucial to the high dechlorination activity of Fe-Pd@PGA NPs.

**Fig. 4:**
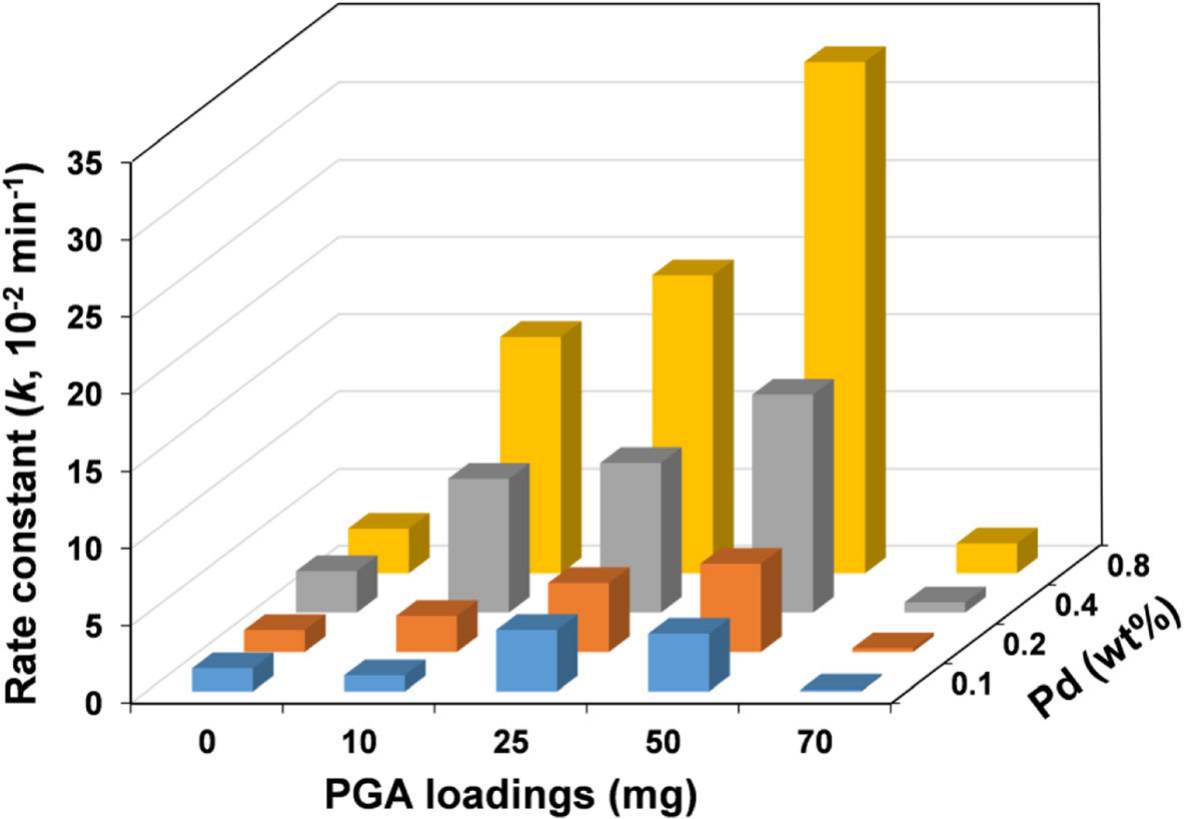
The rate constant of dechlorination of *p*-CP with different PGA and Pd loading

Generally, dechlorination of *p*-CP by Fe-Pd NPs follows a pseudo-first-order rate law [46], which can be modeled using the following equation:

1 $$ \mathit{\ln}\left(\frac{C}{C_0}\right)= kt $$

where *C* and *C*_0_ are chemical concentrations of *p*-CP at times *t* (min) and 0, respectively, *k* (min^− 1^) is the rate constant, and *t* is the reaction time. Therefore, the *k* value of the dechlorination systems is the slope of ln(*C*/*C*_0_) versus *t* over time. Calculations of the rate constant for the dechlorination of *p*-CP with different PGA loadings are shown in Fig. 3d. The k value for the bare Fe-Pd NPs is 0.029 min^− 1^, while it increased to 0.331 min^− 1^ for Fe-Pd@PGA NPs at 50 mg of PGA loading. Figure 4 shows the *p*-CP degradation kinetic rate constants (*k*) of the Fe-Pd NPs with various degrees of PGA and Pd loading. The pseudo-first-order rate constants increased with increasing Pd content, similar to the trend of dechlorination efficiency, and, as PGA loading increased from 0 to 50 mg, *k* increased with increasing PGA loading. However, it appeared that the dechlorination reaction was restrained and thus rate constant showed a decline with a further increase of the PGA loading from 50 to 70 mg. As shown in Additional file 1: Figure S3, the SEM images show the significant aggregation Fe-Pd@PGA NPs due to the excess coverage of PGA, leading to the decrease in dechlorination activity. This decrease in dechlorination activity was also observed in previous studies [47, 48]. Therefore, there was an optimum PGA loading (~50 mg), and the maximum removal reaction rate constant is 0.331 min^− 1^.

Table 1 summarizes the different stabilizers used for nZVI synthesis and the corresponding experimental parameters and results of dechlorination reaction for *p*-CP. We can find that *k* in our work was more than 1 or 2 orders higher than the values reported in previous studies despite of the smaller ratio of Fe to *p*-CP (n_Fe/*p*-CP_) and higher pH value used in this work. In general, a high n_Fe/*p*-CP_ (e.g., > 1000) and neutral or acidic conditions (e.g., pH = 3) were used to achieve high dechlorination activity. In this study, at a n_Fe/*p*-CP_ of 100 and pH 9, Fe-Pd@PGA NPs exhibited high dechlorination activity, which demonstrated that PGA is a promising stabilizer of nZVI for the dechlorination of *p*-CP.

**Table 1:** Comparison of the rate constant (*k*) of dechlorination of *p*-CP in different dechlorination systems

Preparation method	T (°C)	pH	Stabilizer	*wt*% (bimetal)	n_Fe/*p*-CP_	C_0_ (mg/L)	*k* (min^−1^)
Chemical reduction (this work)	25	9	PGA (25 mg)	0.8 (Pd)	100:1	20	19.3 × 10^−2^
			PGA (50 mg)				33.1 × 10^−2^
Ball milling [9]	25	2	–	15 (Ni)	9780:1	20	8.0 × 10^−2^
Chemical reduction [54]	25	3	Mesoporous carbons	2 (Pd)	10:1	50	4.29 × 10^− 2^
Chemical reduction [55]	25	9	CTAB	0.5 (Ni)	1840:1	5	9.3 × 10^−3^
				2.0 (Ni)	920:1		8.6 × 10^−3^
Chemical reduction [56]	30	7	Bentonite	0.5 (Pd)	2300:1	5	1.0 × 10^−2^
				1 (Pd)			6.85 × 10^−2^

### Dechlorination of *p*-CP in the stimulated groundwater

**Fig. 5:**
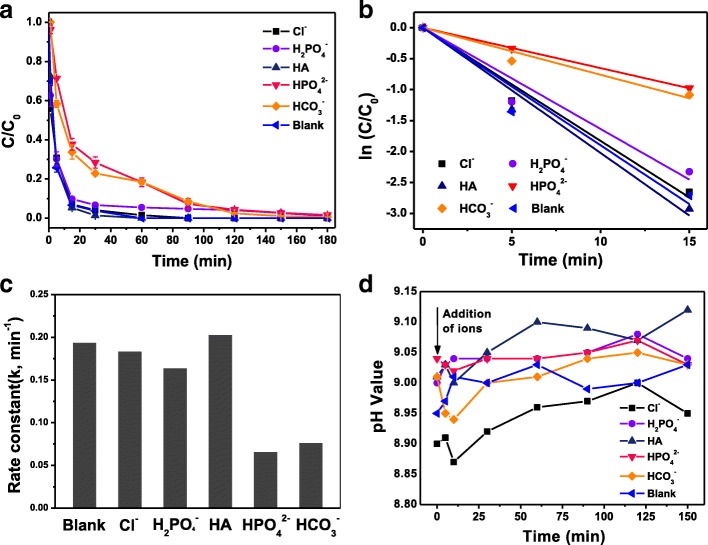
**a** Time courses and (**b**, **c**) rate constant of dechlorination of *p*-CP in the simulated groundwater with different constituents. **d** The changes in pH after the addition of constituents during the reaction

We tested *P*-CP removal in stimulated groundwater to evaluate potential practical applications. The blank experiment was carried out using Fe-Pd@PGA NPs modified with 25 mg of PGA with 0.8% Pd loading. The impact of dissolved constituents in stimulated groundwater was assessed in the presence of four kinds of ions (Cl^−^, H_2_PO_4_^−^, HPO_4_^2−^, and HCO_3_^−^) and HA. As shown in Fig. 5a–c, in the presence of Cl^−^, H_2_PO_4_^−^, or HA, the *k* values are very close to that of the blank experiment, while HPO_4_^2−^and HCO_3_^−^ ions reduce the dechlorination activity, the *k* values decreased from 0.173 to 0.06 and 0.07 min^− 1^. In the previous work, pH increase after the addition of anions was regarded as one of the important reasons for the activity inhibition [49].

The dechlorination reaction process of *p*-CP can be represented as follows [20]:

$$ {\mathrm{Fe}}^0\to {\mathrm{Fe}}^{2+}+2{\mathrm{e}}^{-} $$

$$ 2{\mathrm{H}}_2\mathrm{O}+2{\mathrm{e}}^{-}\overset{\mathrm{Pd}}{\to }2{\mathrm{H}}^{\ast }+2{\mathrm{OH}}^{-} $$

$$ \mathrm{Pd}+{\mathrm{H}}^{\ast}\to \mathrm{Pd}-\mathrm{H} $$

$$ \mathrm{Pd}-\mathrm{H}+\mathrm{R}-\mathrm{Cl}\to \mathrm{Pd}+\mathrm{R}-\mathrm{H}+{\mathrm{Cl}}^{-} $$

The above equations indicate that the pH value is a crucial parameter that affects the degradation rate of *p*-CP. At higher pH, less atomic hydrogen or hydride on the particles’ surface is available to attack the chlorinated molecules. Meanwhile, the ferrous and hydroxyl ions form ferrous hydroxide and precipitate. The surface passivating layer of the ferrous hydroxide and precipitate could hinder the transport of the chlorinated molecules and block the reactive sites on Fe and hence depress the overall reaction rate. With this in mind, we measured the pH values during the dechlorination reaction, as shown in Fig. 5d. The result showed that there is no obvious change in pH values after the addition of different ions, probably due to the presence of PGA. A slight increase in pH values was observed with increasing time, and finally, relatively stable values were observed. The results indicated that pH change is not the dominant reason for ion inhibition. Additionally, we also investigated the dechlorination of p-CP at pH 5 (Additional file 1: Figure S4). The results indicated that weak acid condition was in favor of dechlorination reaction. As for the inhibition of HPO_4_^2−^ and HCO_3_^−^, further study is required to understand the mechanism.

### Mechanism of the Enhanced Dechlorination of *p*-CP by Fe-Pd@PGA NPs

**Fig. 6:**
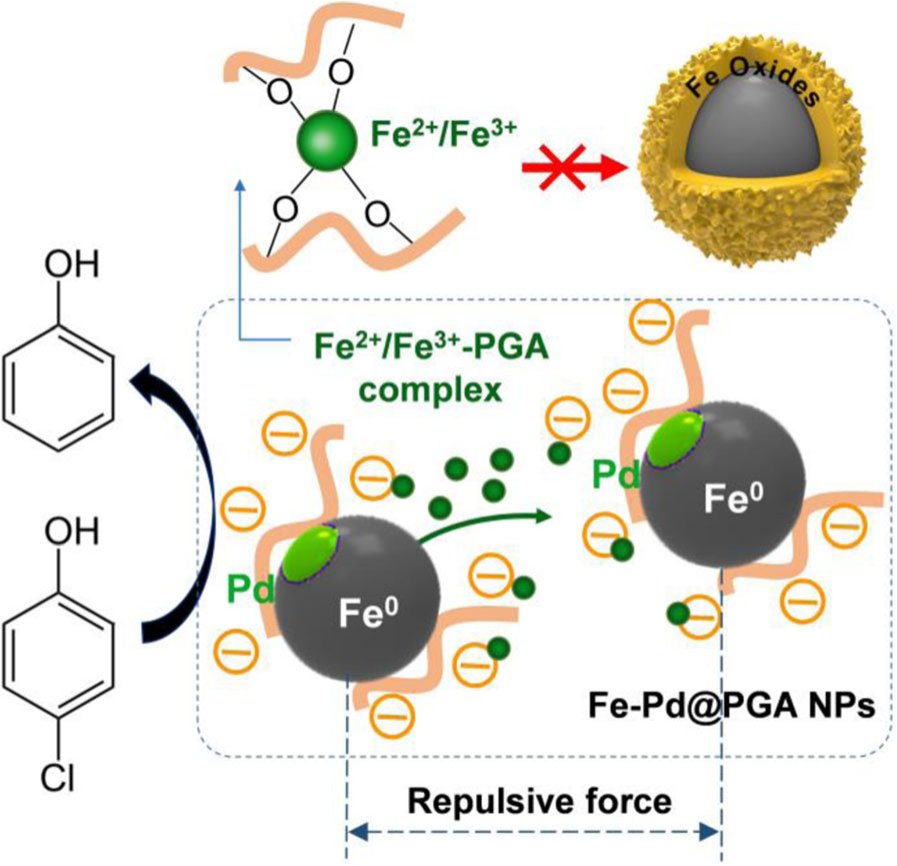
Mechanism of the enhanced dechlorination of *p*-CP by Fe-Pd@PGA NPs

On the basis of our experimental results, a possible mechanism of the enhanced dechlorination by Fe-Pd@PGA NPs was proposed, as shown in Fig. 6.

On the one hand, it is generally assumed that nZVI has iron oxide/hydroxide in the shell [50], with the iron oxide shell contributing positive charges [51]. Therefore, the PGA molecules with abundant anion groups of carboxyl (COO^−^) could easily be attached to the surface of Fe-Pd NPs with positive charges. The carboxyl groups of PGA were inferred to connect with the Fe-Pd NPs’ surfaces through three possible complexation methods: (i) monodentate chelating, (ii) bidentate chelating, and (iii) bidentate bridging [52]. Moreover, the organization of polymeric nanoparticles provides an efficient scaffold with nanoscale dimensions, thus increasing the specific area of the nanoparticles. The modification of Fe-Pd NPs with PGA not only stabilizes the NPs but also prevents the NPs from agglomerating through electrostatic and steric repulsion. Therefore, the excellent dechlorination efficiency and the good dispersity of Fe-Pd@PGA NPs were mainly ascribed to the complex structure of Fe-Pd@PGA NPs and the electrostatic-steric repulsion interaction.

On the other hand, PGA could bind metal ions (e.g., Fe^2+^, Fe^3+^) through chelation and electrostatic binding. As shown in Additional file 1: Figure S5, UV-vis spectra of solutions containing Fe ions and PGA indicated the formation of PGA-iron complexes [53]. During the dechlorination process, Fe^2+^ and Fe^3+^ deposit on the surfaces of Fe NPs, leading to the formation of iron hydroxides and thickening the iron oxide shell. In the presence of PGA, Fe ions coming from the dechlorination reaction would form complexes with PGA and restrain the formation of iron hydroxides, thus preventing the precipitate of Fe hydroxide on the surface, preserving the active sites of the Fe-Pd NPs and providing a favorable microenvironment for retaining dechlorination reactivity. In this case, *p*-*CP* molecules can easily access the active sites and be rapidly dechlorinated by Fe-Pd NPs.

## Conclusions

In summary, we successfully synthesized the Fe-Pd bimetal nanoparticles using PGA as a stabilizer. The as-prepared Fe-Pd@PGA NPs exhibit good dispersity and colloidal stability. The dechlorination performance of Fe-Pd@PGA NPs was evaluated using *p*-CP as the model. The results indicated that PGA significantly promotes the dechlorination of *p*-CP, achieving a high *k* value of 0.331 min^− 1^. This value is much higher than those reported previously despite the smaller ratio of Fe to *p*-CP and higher pH value was used in this work. Furthermore, we found that the Fe-Pd@PGA NPs maintain high activity in the presence of Cl^−^, H_2_PO_4_^−^, and HA, which are generally present in the water. We proposed that the high dechlorination activity of Fe-Pd@PGA NPs is mainly attributed to the electrostatic-steric repulsion interaction and the prevention of Fe hydroxide formation due to the binding ability of PGA to Fe^2+^/Fe^3+^ ions. Given the good stability, high dechlorination activity, and ion tolerance, the Fe-Pd@PGA NPs show promising applications in the dechlorination of chlorinated organic compounds.

## Additional file

Additional file 1: **Figure S1.** SEM-EDS mappings of the Fe-Pd@PGA NPs. **Figure S2.** The suspension of Fe-Pd@PGA NPs standing for one day. **Figure S3.** SEM micrographs of Fe-Pd@PGA NPs with a PGA loading of 70 mg. **Figure S4.** Time courses of dechlorination using Fe-Pd@PGA NPs with the same loadings of PGA (20 mg) and Pd (0.8 wt%) at pH 5 and pH 9. **Figure S5.** UV-vis spectra of complexes of PGA and Fe2+ ions. (a) pH = 7, (b) pH = 9. **Table S1.** Elemental composition of Fe-Pd@PGA NPs with a PGA loading of 50 mg. (DOCX 786 kb)

## Abbreviations

APAM: Anionic polyacrylamide
CMC: Carboxymethyl cellulose
DO: Dissolved oxygen
Fe-Pd@PGA NPs: Palladium-doped zero-valent iron nanoparticles involving poly(γ-glutamic acid)
FTIR: Fourier transform infrared spectroscopy
HA: Humic acid
HPLC: High-performance liquid chromatography
nZVI: Nanoscale zero-valent iron
PAA: Poly(acrylic acid)
PCBs: Polychlorinated biphenyls
*p*-CP: *p*-Chlorophenol
PGA: Poly(γ-glutamic acid)
SEM: Scanning electron microscopy
TCE: Trichloroethylene
TEM: Transmission electron microscopy
XRD: X-ray diffraction
