# Supplementary material for: Poly (γ-Glutamic Acid) Promotes Enhanced Dechlorination of p-Chlorophenol by Fe-Pd Nanoparticles
Source: Nanoscale Res Lett. 2018 Jul 24;13:219. doi: 10.1186/s11671-018-2634-y (PMC6057857; doi:10.1186/s11671-018-2634-y)
Supplement: Supplementary file 1 — Figure S1. SEM-EDS mappings of the Fe-Pd@PGA NPs. Figure S2. The suspension of Fe-Pd@PGA NPs standing for one day. Figure S3. SEM micrographs of Fe-Pd@PGA NPs with a PGA loading of 70 mg. Figure S4. Time courses of dechlorination using Fe-Pd@PGA NPs with the same loadings of PGA (20 mg) and Pd (0.8 wt%) at pH 5 and pH 9. Figure S5. UV-vis spectra of complexes of PGA and Fe2+ ions. (a) pH = 7, (b) pH = 9. Table S1. Elemental composition of Fe-Pd@PGA NPs with a PGA loading of 50 mg. (DOCX 786 kb) [file 11671_2018_2634_MOESM1_ESM.docx]

**Supporting Information**

**Poly (γ-glutamic acid) Promotes Enhanced Dechlorination of *p*-Chlorophenol by Fe-Pd Nanoparticles**

Shiyu Zhang,^1, 2^ Chao Zhang,^2^ Mingyue Liu,^2^ Renliang Huang,^*, 1^ Rongxin Su,^*, 2, 3, 4^

Wei Qi,^2, 3, 4^ Zhimin He ^2^

^1^ Tianjin Key Laboratory of Indoor Air Environmental Quality Control, School of Environmental Science and Engineering, Tianjin University, Tianjin 300072, PR China

^2^ State Key Laboratory of Chemical Engineering, School of Chemical Engineering and Technology, Tianjin University, Tianjin 300072, PR China;

^3^ Collaborative Innovation Center of Chemical Science and Engineering (Tianjin), Tianjin 300072, PR China

^4^ Tianjin Key Laboratory of Membrane Science and Desalination Technology, Tianjin University, Tianjin 300072, P. R. China

* Author to whom any correspondence should be addressed

E-mail: tjuhrl@tju.edu.cn (R. H.), surx@tju.edu.cn (R. S.)

Tel: +86 22 27407799. Fax: +86 22 27407599.

**Supplementary Figures**


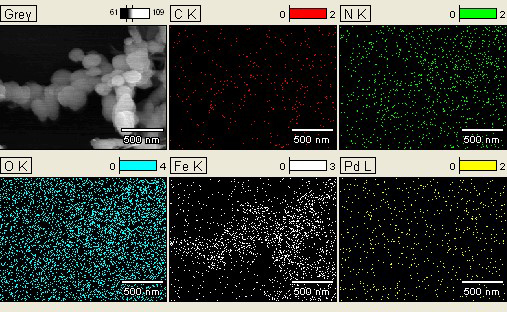


**Fig. S1** SEM-EDS mappings of the Fe-Pd@PGA NPs


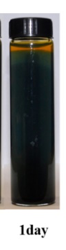


**Fig. S2** The suspension of Fe-Pd@PGA NPs standing for one day.


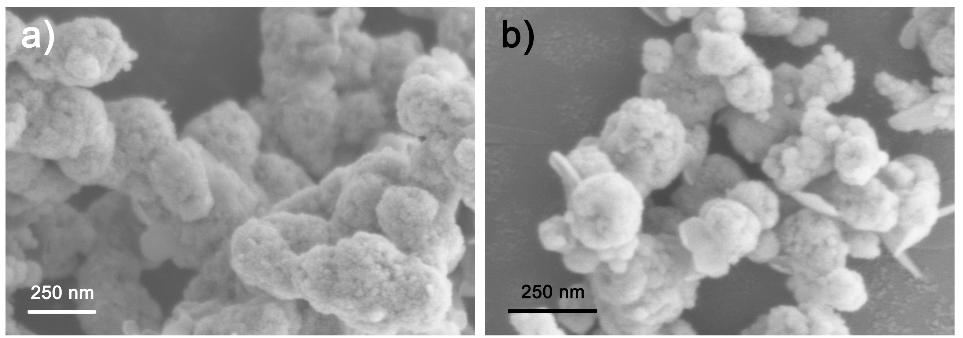


**Fig. S3** SEM micrographs of Fe-Pd@PGA NPs with a PGA loading of 70 mg





**Fig. S4** Time courses of dechlorination using Fe-Pd@PGA NPs with the same loadings of PGA (20 mg) and Pd (0.8 wt%) at pH 5 and pH 9.


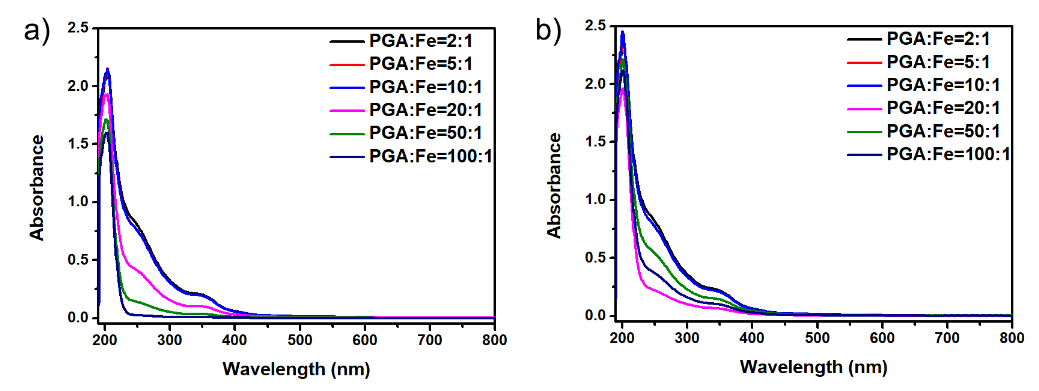


**Fig. S5** UV-vis spectra of complexes of PGA and Fe^2+^ ions. (a) pH=7, (b) pH=9

**Supplementary Tables**

**Table S1** Elemental composition of Fe-Pd@PGA NPs with a PGA loading of 50 mg

| Sample | Composition (%) | | | | |
| --- | --- | --- | --- | --- | --- |
|  | C | N | O | Fe | Pd |
| Fe-Pd@PGA NPs | 10.65 | 3.30 | 52.86 | 32.85 | 0.34 |
